# Anterolateral Thigh Flap Repair of Ruptured Incisional Hernia with Intractable Ascites after Laparoscopic Liver Resection: A Case Report

**DOI:** 10.70352/scrj.cr.24-0124

**Published:** 2025-07-17

**Authors:** Tomohiro Yoshimura, Shinya Hayami, Atsushi Miyamoto, Kensuke Nakamura, Satsuki Tachibana, Atsushi Shimizu, Yuji Kitahata, Masatoshi Sato, Kyohei Matsumoto, Shinichi Asamura, Manabu Kawai

**Affiliations:** 1 Second Department of Surgery, School of Medicine, Wakayama Medical University, Wakayama, Wakayama, Japan; 2 Department of Plastic Surgery, School of Medicine, Wakayama Medical University, Wakayama, Wakayama, Japan

**Keywords:** anterolateral thigh (ALT) flap, incisional hernia, intractable ascites, laparoscopic liver resection, liver cirrhosis

## Abstract

**INTRODUCTION:**

Incisional hernia is one of the postoperative complications after abdominal surgery including laparoscopic liver resection. There is often intractable ascites after liver resection, especially for patients with severe cirrhosis. In the present study, we report the case of ruptured incisional hernia due to the pressure from massive ascites, which was successfully repaired using an anterolateral thigh (ALT) flap.

**CASE PRESENTATION:**

A 78-year-old man had hepatocellular carcinoma and underwent laparoscopic left lateral sectionectomy. There was no short-term postoperative complication during hospital stay and at discharge, but approximately 5 months postoperatively, massive ascites gradually accumulated that was intractable, and resistant to diuretic drugs. There was eventually rupture of incisional hernia at the umbilical port scar, caused by strong compression from this ascites. One year postoperatively, the umbilical skin was seen to be perforated and there was intestinal prolapse. Hernia repair using artificial prosthesis was at risk of infecting ascites and leading to peritonitis. In collaboration with plastic surgeons, we therefore planned incisional hernia repair using an ALT flap. There was severe adhesion between the hernia sac and the small intestine, therefore we had to find the edge of the defective rectus sheath with careful dissection. After resection of the hernia sac, the peritoneum could be closed by continuous suture. The ALT flap obtained by plastic surgeons was elevated through an inguinal subcutaneous tunnel, rotating around the preserved perforator of the lateral circumflex femoral artery. Then, we sutured the edge of the rectus sheath and the ALT skin flap. Operation time was 265 min and the amount of intraoperative bleeding was 15 mL. After the operation, the patient felt dramatic improvement of hernia symptoms and he was discharged on the 16th postoperative day without any complications. Ascites was resolved by use of diuretic drugs and cell-free and concentrated ascites reinfusion therapy.

**CONCLUSIONS:**

Intractable ascites is often a problem in cirrhotic patients after liver resection and can become difficult to treat when complicated by abdominal wall incisional hernias. We successfully performed hernia repair using an ALT flap without the use of artificial materials for ruptured incisional hernia caused by intractable ascites.

## Abbreviations

ALT
: anterolateral thigh
CART
: cell-free and concentrated ascites reinfusion therapy
TFL
: tensor fascia lata

## INTRODUCTION

Laparoscopic liver resection is widely performed for the treatment of hepatocellular carcinoma.^1,2)^ The mortality rate has been decreasing with the improvement of surgical techniques and appropriate perioperative managements in liver resection. However, intractable ascites is sometimes encountered after liver resection, especially in patients with severe cirrhosis.^3,4)^ For refractory ascites following liver resection, treatments such as oral diuretics, branched-chain amino acids, fresh frozen plasma, albumin, and paracentesis followed by CART are considered.^5)^ However, these treatments can cause an increase in medical costs and can prolong hospital stay.^3)^

Incisional hernia is another postoperative complication after abdominal surgery including laparoscopic liver resection. The incidence of umbilical hernias with ascites is reportedly up to 20%.^6)^ A prosthetic mesh is generally used for the treatment of incisional hernia, even in cirrhotic cases.^6,7)^ However, taking into consideration the possibility of intraabdominal infection postoperatively, incisional hernia with ascites is thought to be difficult to treat by prosthetic mesh.^8)^ A definitive treatment strategy for incisional hernia repair with due consideration of the risks of infection has not yet been established. Here, we report a case of ruptured incisional hernia by massive ascites, which was successfully repaired using an ALT flap.

## CASE PRESENTATION

### Present and past history

**Fig. 1:**
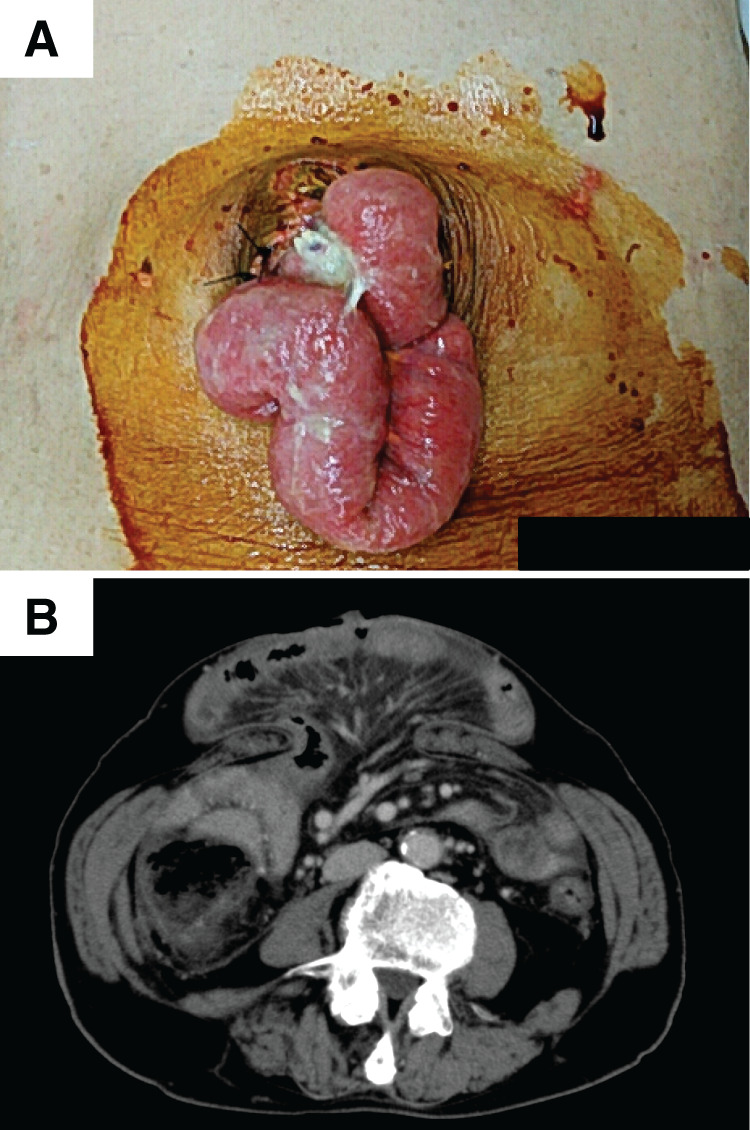
(**A**) Incisional hernia at the umbilical region appeared, caused by the strong compression from ascites. Skin breakdown at the umbilicus led to intestinal evisceration. (**B**) Abdominal computed tomography showing incisional hernia at the umbilical region.

A 78-year-old man had hepatocellular carcinoma derived from alcoholic cirrhosis that was detected in the left lateral section during treatment for ruptured esophageal varices. His past history included hypertension, diabetes mellitus, and stroke, and he had received corresponding medical treatment for each respective disease. His background liver function was good enough for the liver surgery (Child-Pugh score 5A), so he underwent laparoscopic left lateral sectionectomy and was discharged on the 16th postoperative day without short-time complications. However, massive ascites gradually accumulated around 5 months postoperatively that was intractable, and resistant to diuretic drugs. Finally, incisional hernia at the umbilical port scar appeared as a result of strong compression by the ascites. One year after the initial surgery, skin breakdown at the umbilicus led to intestinal evisceration (**Fig. 1A** and **1B**). Simple suturing was first attempted several times in the emergency room to close the wound, but the intra-abdominal pressure was so high that the umbilical skin was broken again. We carefully considered operative indications for this situation. Incisional hernia was evaluated according to previous guidelines^9)^ (**Table 1**). Through the preoperative discussion, we planned incisional hernia repair using an ALT flap in collaboration with plastic surgeons. Liver function was Child-Pugh score 9B; albumin was especially low (**Table 2**). By using CART, 2000 mL of ascites was preoperatively collected, and albumin products were given at 12.5 g/50 mL per day for 3 days before surgery. Moreover, C-reactive protein was high; therefore, cefazolin was used perioperatively as antibiotic prophylaxis.

**Table 1:** Datasets for incisional ventral hernia (Modified from previous guideline^9)^)

	Definition	Evaluation
Preoperative variables		
Age	Years since birth	79
Sex	M; F	M
BMI	Weight (kg)/height^2^ (m^2^)	20.7
COPD	Diagnosis of COPD	No
Smoker	EuraHS6 (never smoker, ex-smoker > 12 months; occasional smoker; daily smoker)	Ex-smoker >12 months
Diabetes (types 1 or 2)	Diagnosis of type 1 or 2 diabetes	Type 2 diabetes
Immunosuppression/steroids	Diagnosis requiring immunosuppression or chemotherapy	No
ASA fitness grade	ASA fitness grade	2
Hernia variables		
Hernia width	Maximum defect width; EHS classification	6 cm
Loss of domain	Volumetric measurement: Sabbagh method	Not calculated
European Hernia Score	EHS classification; incisional hernia	M3W2
No. of hernia defects	No. of defects in anterior abdominal wall	1
Divarification	Separation >2 cm between rectus muscles	No
Reducibility	Reducible; irreducible, no skin changes; irreducible, with skin changes; irreducible, causing bowel obstruction	Irreducible, with skin changes
Previous abdominal operations	No. of midline, subcostal, and transverse incisions	Single umbilical incision and five trocar sites incisions in the upper abdomen
No. of previous hernia repairs ± mesh	No. of previous hernia repairs and mesh	No
Previous wound infection (SSI)	Previous SSI at site of hernia repair	No
Hernia defect area	Defect area where hernia sac passes through abdominal wall	23.5 cm^2^
Stoma present	Abdominal wall ostomy present	No
Previous component separation	Previous anterior component/transversus abdominis release	No
Current mesh infection	Chronic infection, sinus or abscess at location of mesh	No
Perioperative variables		
Mode of surgery	Mode of surgery (laparoscopic; open; robotic)	Open
Mesh or suture	Method of repair	Flap reconstruction
Ventral Hernia Working Group assessment	Low risk; comorbid; contaminated; dirty	Comorbid
CDC assessment	Clean; clean-contaminated; contaminated; dirty	Clean-contaminated
Preoperative botulinum toxin	Preoperative injection of botulinum toxin to strap muscles	No
Component separation	Anterior component/transversus abdominis release	No
Concomitant gastrointestinal procedure	Bowel resection; cholecystectomy; stoma formation	No
Mesh repair		Evaluation about flap reconstruction
Exact mesh name	Trade name and flap type	Anterolateral thigh flap
Flap fixation technique	Sutures or tacks (absorbable; non-absorbable)	Sutures (absorbable)
Position of mesh	ICAP nomenclature	N/A
Mesh size	Intraoperative measurement	The size of skin flap and fascia lata were planned as 7 × 22 and 12 × 22 cm.
Bridging versus fascial closure	EHS definitions. Anterior fascia completely closed or not completely closed	N/A
Mesh overlap	Mesh overlap area/defect area ratio. Ellipse: Overlap = πAB − πab	N/A
Suture repair		
Suture type	Absorbable or non-absorbable material used	Absorbable material used
Postoperative outcomes		
SSI	CDC definition of SSI: a) Superficial; b) deep; c) organ space	No
SSO	Any adverse wound event. SSI, seroma, hematoma, fistula, etc.	No
SSO requiring intervention	SSOs requiring procedural intervention	No
Mesh infection	Chronic infection, sinus or abscess at location of mesh	No
Chronic pain	Pain lasting longer than 3 months after surgery	No
Hernia recurrence	EHS definition: a protrusion of the contents of the abdominal cavity or preperitoneal fat through a defect in the abdominal wall at the site of a previous repair of an abdominal wall hernia	No
Clavien–Dindo grade	Grades I–V. Grade IIIb: intervention under general anesthesia	No
30-day reoperation rate	Abdominal operation under general or regional anesthesia within 30 days of primary ventral hernia repair	No

ASA, American Society of Anesthesiologists; BMI, body mass index; CDC, Centers for Disease Control and Prevention; COPD, chronic obstructive pulmonary disease; EHS, European Hernia Society; EuraHS, European Registry of Abdominal Wall Hernias; F, female; ICAP, International Classification of Abdominal Wall Planes; M, male; N/A, Not available; SSI, surgical site infection; SSO, surgical-site occurrence

**Table 2:** Laboratory data on admission

Value	(Unit)	Value	(Unit)
WBC	54.6 (10^2^/μL)	Alb	2.3 (g/dL)
RBC	352 (10^4^/μL)	AST	25 (IU/L)
Hb	11.5 (g/dL)	ALT	19 (IU/L)
Ht	34.2 (%)	ALP	206 (IU/L)
PLT	16.5 (10^4^/μL)	T-bil	0.7 (mg/dL)
Neu	77.9 (%)	D-bil	0.2 (mg/dL)
Ly	12.5 (%)	Cre	1.02 (mg/dL)
CRP	14.79 (mg/mL)	eGFR	54.2
AFP	1.6 (ng/mL)	BUN	25.3 (mg/dL)
AFP-L3%	Undetectable (%)	Na	137 (mEq/L)
PIVKA-II	16 (mAU/mL)	K	4.5 (mEq/L)
HA	100 (ng/mL)	PT (ratio)	86.0 (%)
HbA1c	6.4 (%)	PT-INR	1.10

AFP, alpha-fetoprotein; AFP-L3%, lectin-reactive alpha-fetoprotein; Alb, albumin; ALP, alkaline phosphatase; ALT, alanine aminotransferase; AST, aspartate aminotransferase; BUN, blood urea nitrogen; Cre, creatinine; CRP, C-reactive protein; D-bil, direct bilirubin; eGFR, estimated glomerular filtration rate; HA, hyaluronic acid; Hb, hemoglobin; HbA1c, glycated hemoglobin; Ht, hematocrit; K, kalium; Ly, lymphocyte; Na, natrium; Neu, neutrophil; PIVKA-II, protein induced by vitamin K absence or antagonist-II; PLT, platelet; PT, prothrombin time; PT-INR, international normalized ratio of prothrombin time; RBC, red blood cell; T-bil, total bilirubin; WBC, white blood cell

### Surgical procedures

**Fig. 2:**
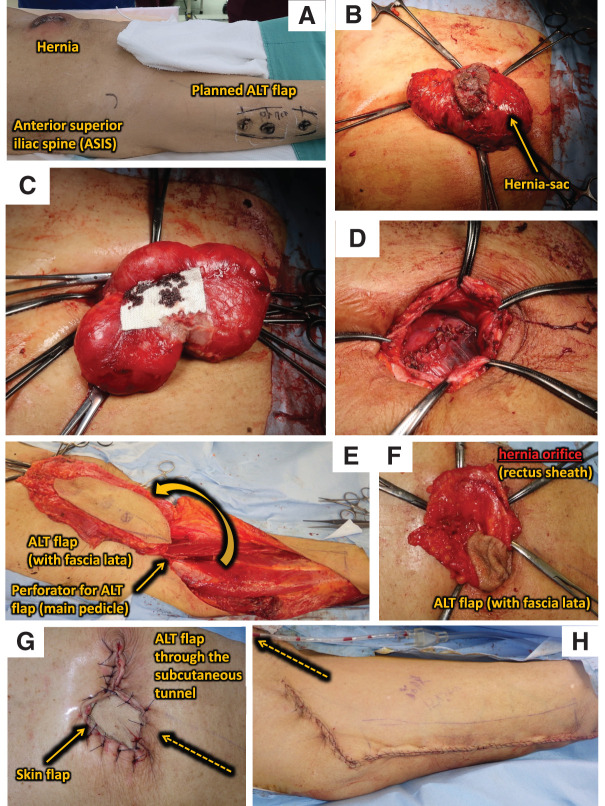
Surgical procedures. (**A**) Preoperative planning. (**B**) Resection of the hernia-sac. (**C**) After resection of the hernia-sac, the small intestine is returned to the abdominal cavity. (**D**) Peritoneal closure and identification of hernia orifice. (**E**) ALT flap planning. (**F**) Elevation of an ALT flap through inguinal subcutaneous tunnel. (**G**) Completed picture (abdomen). (**H**) Completed picture (leg).

First, as preoperative planning, the locations of an ALT flap and incisional hernia were marked (**Fig. 2A**). A spindle-shaped skin incision was made and the hernia sac was identified. After careful dissection between the small intestine and the greater omentum for adhesion to the hernia sac without injuring the bowel, we resected the redundant hernia sac (**Fig. 2B** and **2C**). A fascia defect was measured as 5 × 6 cm and the peritoneum was closed by continuous suture using 2-0 Coated Vicryl (Johnson and Johnson, Tokyo, Japan) (**Fig. 2D**). Then, ALT flap preparation was started by plastic surgeons (**Fig. 2E**). The size of the skin flap and fascia lata were planned as 7 × 22 and 12 × 22 cm, respectively. Perforators from the descending branch of the lateral femoral circumflex artery were carefully preserved. The ALT flap was rotated around the most cranial perforator. Inguinal skin was not incised and a subcutaneous tunnel was made. The ALT flap was elevated through the inguinal subcutaneous tunnel after de-epithelialization (**Fig. 2E**–**2H**) and fixed by interrupted suture using 1-0 PDS plus (Johnson and Johnson) with rectus abdominis fascia and 2-0 nylon with skin. Subcutaneous closed suction drain (SB drain 5 mm; SB-KAWASUMI LABORATORIES, Kanagawa, Japan) was inserted at both the reconstruction and flap donor sites. Operation time was 265 min and the amount of intraoperative bleeding was 15 mL.

### Postoperative management

**Fig. 3:**
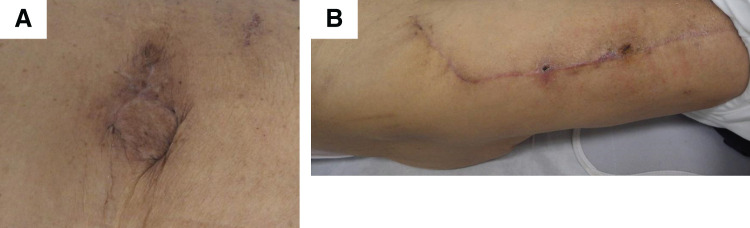
Wound 3 months after this surgery. (**A**) abdomen. (**B**) right leg.

After the operation, the patient felt his hernia symptoms had dramatically improved. He was given albumin 12.5 g/50 mL/day also for 3 days after the surgery and discharged on postoperative day 16 without any complications. Ascites disappeared by use of diuretic drugs and 3 rounds of CART. The wound was in good condition at 3 months after surgery (**Fig. 3A** and **3B**) and 36 months later there has been no recurrence of incisional hernia since the hernia repair surgery.

## DISCUSSION

We successfully performed hernia repair using an ALT flap and without artificial materials for ruptured incisional hernia caused by intractable ascites. For this patient, it was necessary to treat the conflicting conditions, both incisional hernia and massive intractable ascites, simultaneously or sequentially. However, these 2 conditions required somewhat contradictory treatment because hernia repair surgery can only be performed after management of ascites and because using artificial prostheses risks infecting the ascites and possibly leading to peritonitis.

Incisional hernia is one of the potential postoperative complications after abdominal surgery including laparoscopic liver resection. Ascites is another potential postoperative complication after liver resection, with frequency reported at about 25.5%.^10)^ Intractable ascites is sometimes seen, especially in patients with more severe liver cirrhosis, and massive ascites with incisional hernia might result in abdominal wall rupture due to excessive compression.^11)^ Hernia repair must remain tension-free,^12)^ and inadequate control of ascites may lead to recurrence due to increased intra-abdominal pressure, as observed in this case. To keep the wound tension-free, prosthetic mesh is generally used for the treatment of incisional hernia,^13)^ however, mesh infection after abdominal wall hernia repair is a life-threating complication.^14)^ Hernia mesh repair with liver cirrhosis and ascites has been reported as a safe approach with a low risk of infection;^11,13)^ on the other hand, there is also a report about abdominal wall repair without mesh in conditions of suspected infection.^15)^ The use of artificial products is therefore controversial, and incisional hernia with ascites may be difficult to treat by prosthetic mesh. Hernia repair using artificial prosthesis has a risk of infection with ascites and may lead to peritonitis. Adaptation of the Denver shunt was also considered for ascites control, but hernia repair may be difficult because Denver shunt should be avoided in conditions of suspected infection by ruptured incisional hernia.^16)^

Previously, we used a pedicled TFL flap for reconstruction of an infected recurrent ventral hernia after a mesh repair.^17)^ We therefore decided to consult with plastic surgeons and planned incisional hernia repair using a flap. Due to its muscular component and relatively greater thickness, the TFL flap is less appropriate for small-volume reconstruction. Furthermore, its use is associated with a higher risk of donor site morbidity compared with the ALT flap. Therefore, the ALT flap was selected as the more suitable option in this case. ALT flap, first reported by Song et al. in 1984, is a large flap harvested from the thigh with a long neurovascular pedicle.^18)^ Blood flow of the flap was based on the descending branch of the lateral circumflex femoral artery. ALT flaps are commonly used in abdominal wall reconstruction.^19,20)^ Reconstruction with an ALT flap appears to be useful when abdominal wall reconstruction with an artificial prosthesis is difficult due to infected ascites.

## CONCLUSIONS

Intractable ascites is often a problem in patients after liver resection and may be difficult to treat when complicated by abdominal wall incisional hernias. We successfully performed hernia repair using an ALT flap without the use of artificial materials such as mesh for ruptured incisional hernia with intractable ascites.

## ACKNOWLEDGMENTS

We acknowledge proofreading and editing by Benjamin Phillis at the Clinical Study Support Center at Wakayama Medical University.

## DECLARATIONS

### Funding

The authors have no financial support to declare.

### Authors’ contributions

Study conception and design: TY, SH

Acquisition of data: TY, SH

Analysis and interpretation of data: TY, SH, KM

Drafting of manuscript: TY, SH

Critical revision: AM, KN, ST, AS, YK, MS, KM, SA, MK

All authors have read and approved the manuscript, and they are responsible for the manuscript.

### Availability of data and materials

De-identified patient data that support the findings of this case report are available upon reasonable request from the corresponding author.

### Ethics approval and consent to participate

This work does not require ethical considerations or approval. Informed consent to participate in this study was obtained from the patient.

### Consent for publication

Informed consent was obtained from the patient for the publication of this case report.

### Competing interests

The authors declare that they have no competing interests.
